# Content Analysis of Three-Dimensional Model Technologies and Applications for Construction: Current Trends and Future Directions

**DOI:** 10.3390/s24123838

**Published:** 2024-06-13

**Authors:** Nhien Le, Daniel Tran, Roy Sturgill

**Affiliations:** 1 Department of Civil, Environmental, and Architectural Engineering, University of Kansas, Lawrence, KS 66045, USA; daniel.tran@ku.edu; 2 Civil, Construction and Environmental Engineering Department, Iowa State University, Ames, IA 50011, USA

**Keywords:** three-dimensional models, meta-analysis, augmented reality (AR), virtual reality (VR), computer vision, sensing, laser scanning

## Abstract

The proliferation of digital technologies is substantially transforming inspection methodologies for construction activities. Although the implementation of a three-dimensional (3D) model has emerged as an advantageous, feasible inspection application, the selection of the most suitable 3D models is challenging due to multiple technology options. The primary objectives of this study were to investigate current trends and identify future technologies for 3D models in the construction industry. This study utilized systematic reviews by identifying and selecting quality journals, analyzing selected articles, and conducting content analysis and meta-analysis to identify dominant themes in 3D models. Results showed that the top technologies used to model construction projects are building information models, remote sensing, stereo vision system/photo processing programs, and augmented reality/virtual reality. The main benefits and challenges of these technologies for modeling were also determined. This study identified three areas with significant knowledge gaps for future research: (1) the amalgamation of two or more technologies to overcome project obstacles; (2) solution optimization for inspections in remote areas; and (3) the development of algorithm-based technologies. This research contributes to the body of knowledge by exploring current trends and future directions of 3D model technologies in the construction industry.

## 1. Introduction

Despite the prevalent utilization of two-dimensional (2D) models, previous research has revealed several deficiencies in these models during construction activities [1]. In fact, conventional (2D) models of construction projects are now being substituted, at least partially, by a variety of digital transformations [2,3,4]. In contrast, the three-dimensional (3D) model has proven to be an effective and efficient tool to enhance the interactivity and practicality of a construction project [5]. A 3D construction model provides specific project information, thereby improving the productivity of a project’s survey, design, construction, and maintenance. Precise and timely construction modeling approaches may offer confidence that projects reasonably conform to specifications [6,7]. In addition, 3D models may fulfill specific criteria to meet project expectations and specifications throughout the construction process, including surveying, planning, arranging resources, constructing, and inspection [8,9].

A 3D model derived from the analysis of historical databases is a promising option to mitigate inspection costs for building structures [10,11,12]. One study [13] developed a building information model (BIM) content management system to improve productivity by extracting content from more than 30,000 technical BIM 3D views, including plans, sections, and details of historical projects. The unsupervised association rule mining model then uses these BIM views to explicate associative relationships among BIM objects and effectively predict content needs, saving time in conventional BIM workflows and eliminating the need to collect diverse data, such as user similarity and personalized ratings, resulting in significant long-term savings. Additionally, the use of technologies to generate 3D models can potentially offset workforce deficiencies related to construction project inspections [14,15,16,17].

New technologies such as augmented reality (AR) [18], virtual reality (VR) [19], computer vision [20], laser scanning [21], and extensive data analysis [22] are increasingly utilized to facilitate real-time information access and improve data collection accuracy. However, the utilization of these emerging technologies and their corresponding applications in construction may lead to perplexity and incompatibility in project development [23]. For example, the use of BIM in the construction industry has exposed a lack of implementation familiarity, unclear roles and responsibilities, and insufficient legal frameworks to integrate owner perspectives in design and construction [24]. Although 3D model technology has been well established with numerous applications, minimal research has examined and summarized the systematic use of these modeling technologies for the construction industry. In addition, the available information regarding the use of these technologies for 3D models is fragmented, scattered, and unevaluated. Therefore, this study conducted a systematic review and content analysis of relevant documents published in the last two decades to identify current and future 3D modeling trends for the construction industry [7]. This study also investigated the capacities, requirements, advantages and disadvantages, and other related issues of 3D modeling technology.

## 2. Background

Engineers typically use conventional or 2D drawings as a reference for construction projects. However, these drawings may lack precision and detail of construction processes [2,25], and sharing and collaboration of physical drawings may be cumbersome, especially when the project has multiple stakeholders at different construction sites. Conventional drawings are also often difficult to integrate with modern software tools, making it more challenging to perform quantity takeoffs, cost estimation, and clash detection [1]. These challenges may be resolved using 3D models or 3D digital representations of a building or structure using specialized software and tools [21]. In the construction industry, 3D modeling involves the creation of digital representations of physical structures or environments using specialized techniques. These detailed and accurate models are valuable tools for architects, engineers, and construction professionals as they allow for visualization, analysis, and simulation of various aspects of a project before construction begins. Furthermore, these models simulate the design, construction, and operation of a project in a virtual environment, enabling users to view, interact with, and manipulate them on a computer or other compatible devices.

Traditionally, project owners have specified quality standards, including detailed instructions about required materials and construction methods, and employed on-site staff for the construction process. Although this traditional approach typically provides owners with quality products, engaging in on-site discussions to address field issues is time-consuming and costly. Comparatively, the advancement and interactivity of 3D models on computers or mobile devices save time and reduce the cost of travel to construction sites [10,15,26]. Users can also beneficially zoom in, rotate the model on multiple axes, and pan across models to explore perspectives and gain a comprehensive understanding of each structural element. A previous study [27] highlighted the benefits of employing 3D-engineered models in highway construction projects to improve communication, constructability, and production. The use of 3D-engineered models is foundational to the integration of other advanced technologies, such as automated machine guidance, clash detection, and quantity verification, that can substantially impact construction staffing requirements.

The integration of cutting-edge technologies such as BIM, stereo vision systems, photogrammetry, and 3D scanning in construction processes has revolutionized the industry, leading to the widespread adoption of digital tools and technologies across every stage of construction, including design, construction, operations, and inspections. These technologies are interconnected in various ways; for instance, photogrammetry utilizes photographs to create 3D models, while 3D scanning captures precise point cloud data of physical structures. Their collective impact is instrumental in digitalizing the physical environment and seamlessly incorporating real-world context into the BIM environment [18]. These technologies are becoming commonplace in construction due to their potential to improve construction activities while improving the efficiency and productivity of the construction process. For example, technologies and digital tools help ensure the accuracy and validation of material testing and inspection processes.

## 3. Research Method

**Figure 1:**
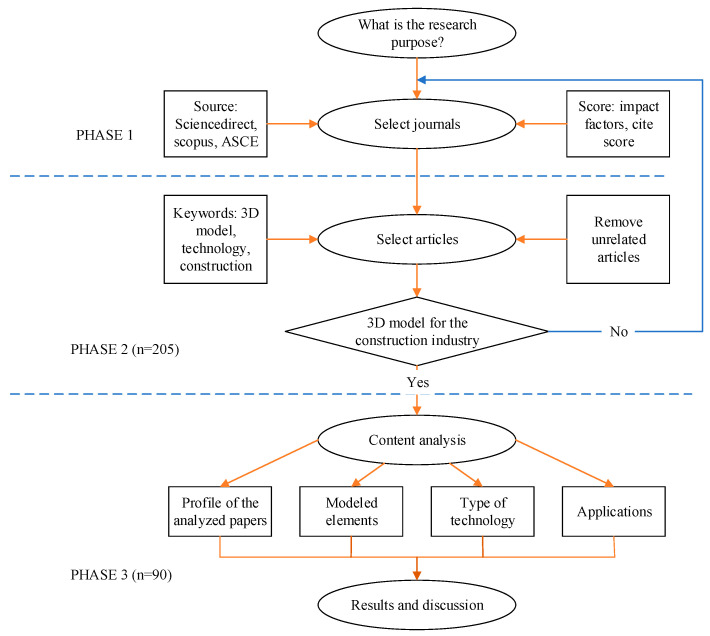
Research method.

The objective of this study was to investigate the current and future trends of 3D modeling technology in the construction industry. The authors employed a systematic review following structured Preferred Reporting Items for Systematic Review and Meta-Analyses (PRISMA) guidelines to ensure the clarity and comprehensibility of systematic review and meta-analysis reporting, including procedure development, search strategy, data extraction, and data synthesis. Figure 1 shows the three phases of the study’s research method: identifying and selecting quality journal articles, analyzing the selected articles, and conducting content analysis and meta-analysis.

In the initial stage of the research, the focus was on defining the research objectives and choosing reputable journals with a high impact and a minimum CiteScore of 0.7 [28]. Stringent criteria were applied to ensure that only top-quality studies were considered for the review. In the second phase of the research, the authors selected related articles using keywords such as “3D models”, “design”, “construction inspection”, “modeling”, and “construction technology”, excluded irrelevant studies, and removed duplicated articles. In the third phase, the authors used NVIVO software (version 11) to manage, analyze, and visualize qualitative data. A total of 205 articles were exported from Zotero (v. 6) and imported to NVIVO, and then the data were coded based on technologies and applications in the construction field. The rigorous coding process included reviewing abstracts, research methodologies, conclusions, and key findings and highlighting and assigning the selected texts or multimedia to appropriate nodes [7,28]. The authors also added comments to provide additional context or insight. Finally, the authors extracted the nodes, sources, and references and generated relationships throughout the coded data.

**Figure 2:**
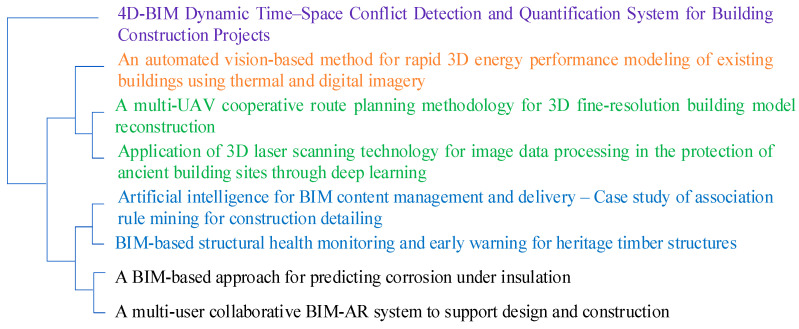
Representative cluster analysis.

In this research, we conducted a comprehensive examination of the qualitative data using advanced analysis techniques such as word frequency analysis and cluster analysis functions (Figure 2). The word frequency analysis allowed us to systematically identify and quantify the most commonly used words and phrases within the qualitative data. This approach helped us pinpoint specific areas of interest or concern among the study participants by focusing on the frequency of particular words. Additionally, the cluster analysis enabled us to identify and group similar articles, themes, or concepts, unveiling patterns and relationships that may not have been immediately apparent. The results highlighted 90 articles from 21 journals, which were cross-checked to ensure validity. A comprehensive analysis was then carried out once the articles were determined and characterized. In addition to examining and analyzing 90 articles, several technical reports from the Federal Highway Administration (FHWA) and National Highway Research Programs (NCHRP) were selected to assess and contrast the practical implications of 3D models. This structured approach, guided by the PRISMA framework, ensured a robust and systematic examination of the literature, enhancing the reliability and validity of the findings.

Figure 2 illustrates the process of utilizing context similarity to generate meaningful clusters in the content analysis of 3D modeling technology. By calculating contextual similarities, documents exhibiting similar relative word patterns are grouped together using a clustering algorithm. The detailed results of this method are presented in the results section, providing insights into the various technologies employed for 3D modeling.

## 4. Profile of Selected Articles

Table 1 presents the initially selected articles, analyzed articles, CiteScores, and impact factors. A total of 21 journals were selected based on CiteScores and impact factors that ranged from 3.5 to 20.9 and 2.24 to 11.77, respectively. Articles from Automation in Construction (AC), the International Society Journal of Photogrammetry and Remote Sensing (ISPRS), the Journal of Computing in Civil Engineering (JCCE), and the Journal of Construction Engineering and Management (JCEM) were analyzed to identify technologies used for construction activities.

Table 2 provides a summary of the primary technologies commonly employed for 3D modeling in the construction industry. This information has been derived from an initial review of relevant articles utilizing advanced analysis methods such as word frequency analysis and cluster analysis. The articles were ordered by the frequency of the mention of a 3D model in each article; a total of 371 frequencies were summarized using PRISMA guidelines. The most common technologies used for 3D modeling were BIM (20.8%), remote sensing (19.1%), stereo vision/photo processing programs (15.4%), AR/VR (9.2%), deep learning, reinforcement learning (7.3%), robots (4.3%), and unmanned aerial vehicles/systems (UAVs/UASs) (4.0%).

**Figure 3:**
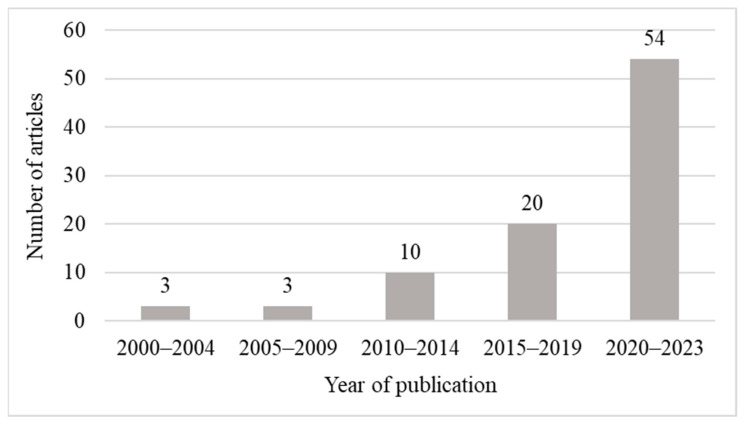
Summary of analyzed articles by year (*n* = 90).

Figure 3 illustrates the distribution of the 90 analyzed articles by year of publication in 5-year intervals, except for the last interval of 4 years. The number of studies substantially increased over the last decade, reflecting the trend across the 205 selected articles. Most of the examined articles (60%) were published between 2020 and 2023, thereby highlighting the recent significance of 3D models for the construction industry. As shown in Table 1, most of the articles were derived from the AC (32.2%), ISPRS (17.8%), JCCE (10.0%), JCEM (8.9%), and AEI (5.6%).

Table 3 lists the eight most prevalent elements for 3D models as determined from the review of 90 articles. The utilization of 3D models for buildings emerged as the dominant element, accounting for the highest proportion of use at 48%. Based on previous research [29] that highlighted the high proportion of buildings (exceeding one-third) that contribute to energy demand worldwide, the findings of this research are appropriate and correspond with current demands related to energy consumption, historical building reconstruction, and preservation.

Three-dimensional models can be used to offer a multitude of possibilities for visualizing and creating detailed representations of urban infrastructure and natural environments [32,33]. They can be used to accurately depict streets, tree distributions, building layouts, and the complex surfaces of unstructured areas. Furthermore, these models are valuable for optimizing and validating the designs and operations of critical infrastructure such as dams, tunnels, roads, and bridges, showcasing the breadth of their utility across diverse engineering and design disciplines [17]. An in-depth analysis provided in the study [17] highlights that the ongoing project focuses on the requalification and enhancement of a specific section of the SS 245 road located in northern Italy. The primary aim of this initiative is to eliminate the railroad crossing along the Castelfranco–Bassano railway line. Notably, a comprehensive 3D model has been successfully developed for a two-lane roundabout and a solid road using “Autodesk AutoCAD Civil 3D” and “Revit Structure”.

## 5. Results and Discussion

Previous researchers have identified an increasing need for technology to improve the efficiency and accuracy of increasingly complex construction processes [15,19,42,43]. This section categorizes technologies used for 3D modeling in the construction industry into five classes: visualization and imagery, geospatial, algorithm-based, automated, and software. However, this classification process may vary based on the unique definitions, goals, and functionalities of specific technologies [7,28]. Different 3D model technologies are designed with particular purposes, which affects how they should be classified.

### 5.1. Visualization and Image Modeling Technologies

Table 4 summarizes six common visualization and image modeling technologies for 3D models based on the review of 90 articles. These six common technologies are classified under the category of visualization and image modeling technologies due to their shared ability to generate, manipulate, and enhance visual representations of 3D objects and environments. Image modeling technologies, in addition, involve creating accurate 3D models from various types of input data, such as photographs, stereo vision systems, or other digital images. BIM was shown to be increasingly popular in the construction industry because it advantageously creates and manages digital representations of places or objectives, which saves time and money and enhances communication and coordination among project team members [44]. BIM also allows users to analyze and improve project factors such as performance, safety, and constructability [45]. The United States has been at the forefront of advancing BIM within the construction industry. As early as 2003, the General Services Administration (GSA) launched the “National 3D-4D program” with the objective of progressively integrating 3D, 4D, and BIM technologies into all significant public projects [45]. Furthermore, in 2007, the GSA mandated the use of BIM for spatial program validation across all of its projects. Furthermore, BIM can provide geometric and semantic information for building surfaces to aid construction inspection activities [17,46,47,48].

Using cluster analysis (Figure 2), this research found that a combination of hybrid approaches may generate 3D models, such as integrating UAVs/UASs and BIM [49,50], building condition risk assessment models and BIM [10], BIM and AR [18], automated facility inspection using robotics and BIM [51], BIM with machine learning and computer vision techniques [52], and integrating RFID data into BIM [48]. Although BIM was developed to model buildings, its use is expanding to include environmental infrastructure (e.g., dams, levees, and embankments), utility infrastructure (e.g., pipelines for gas, water, and sewage), energy infrastructure (e.g., power generation plants, oil, and gas), and transportation infrastructure (e.g., roads, railways, and bridges) [17]. Algorithms are currently being developed to analyze BIM data systematically in order to detect safety hazards during construction inspections and address other industry concerns [53]. The automated safety checking platform alerts construction engineers and managers about necessary safety measures from the Occupational Safety and Health Administration (OSHA) to prevent fall-related accidents before construction begins. The study [53] presented a case study of implementation in Tekla, demonstrating a construction project during the construction process. The identified openings have various sizes and geometric shapes (polygonal, rectangular, and circular) that pose potential fall hazards. The developed rule-checking system successfully detected holes and edges of slabs 100% of the time. The default prevention equipment (guardrail system and cover) is automatically applied, demonstrating the robustness and scalability of the implemented algorithms.

**Table 4:** Visualization and image modeling technologies for 3D models.

No.	Technology	Description and Typical Application	Key References	Number of References
1	BIM	BIM creates a shared digital model for a building or infrastructure project, which allows for a more efficient and accurate design and construction process, helps reduce errors, avoids conflicts, and generates accurate cost estimates and construction schedules.	[18,24,26,35,50,52,54,55,56,57,58]	11
2	Stereo vision system	A stereo camera may provide an entire field of view for 3D measurement in an unstructured and dynamic environment. It is widely used in different areas, including people tracking and mobile robotic navigation.	[59,60,61,62,63,64]	6
3	High-resolution satellite imagery	Satellite imagery is an essential tool for the visualization of urban planning, infrastructure, and disaster responses.	[65,66,67,68,69]	5
4	Photo processing programs	Photogrammetry has considerable value in cultural heritage applications because it enables accessibility through 3D modeling and expedites archaeological documentation.	[70,71,72,73,74]	5
5	Topological model	Topological models are mathematical models used in fields such as physics, computer science, and construction modeling to simplify the complexity of construction design and provide a more precise and intuitive visualization of the overall structure.	[75,76,77]	3
6	Mesh simplification	Mesh simplification is a modeling technique to reduce model complexity and decrease the amount of required memory for display.	[40,78,79]	3

As described in Table 4, stereo vision is an imaging technique based on the triangulation of rays from multiple viewpoints, similar to how human vision perceives 3D objects, while high-resolution satellite imagery is an efficient tool to enhance understanding of the Earth and improve decision-making about resource usage and is available for nearly every geographic location on Earth from multiple viewpoints [67]. The study highlighted an important issue in practical applications where digital surface models (DSMs) often lack accuracy for high-resolution tasks like 3D city modeling due to noise. To address this, the researchers introduced RESDEPTH, a convolutional neural network (a subset of deep learning) that can learn detailed geometric information from example data. They conducted simulations using diverse and geographically distributed training samples, such as high-quality data from different regions within a country, to train a joint model for all regions of interest. This approach involved sampling training data from five regions in Berlin and Zurich to enhance the model’s performance. A study [72] demonstrated that photometric stereo techniques can effectively recover the surface normal of 3D objects illuminated from different directions. The photometric stereo method may be applied in the field of construction, where accurate surface detail is essential for creating precise models of building materials (concrete, metal, and glass surfaces) and other structures. They also indicated that data-driven photometric stereo methods, which leverage machine learning and deep learning techniques, have shown a significant performance advantage over traditional approaches. Table 4 also includes photogrammetry, which is valuable for creating 3D models and expediting the documentation of archaeological sites [68] and topology models [76]. Finally, mesh simplification, or decimation, is a crucial process that optimizes 3D representations of structures or models [79].

However, the use of visualization and image modeling technologies for construction projects can be challenging (Table 5). Although BIM has proven to be an effective tool for building models, some fused BIM reconstruction techniques are limited because they rely on collected data, such as the depth of the camera [24,80], meaning the generated semantic-rich 3D map may be missed and incomplete due to the camera speed as it collects data. The lack of a sufficient legal framework to integrate owner views in design and construction with BIM is also a disadvantage. The inadequacy of laws, regulations, and guidelines may derail building and infrastructure project designs and construction processes.

Previous researchers have also found that individual techniques may not be ideal for modeling sophisticated, intricate structures of buildings and surfaces [61,64,67,89]. One study [89] utilized a hybrid data modeling strategy to erect neighborhood surface patches and reconstruct a building model, successfully modeling the topological relationship between surface patches. Similarly, another study [13] developed an artificial intelligence (AI)-backed BIM content recommender system to improve BIM productivity using data from hundreds of historical projects. Instead of collecting data regarding user preferences, ratings, or individualized information, their approach used established BIM standards with consistent technical details, proving the efficacy of the amalgamation of two or more of these technologies.

### 5.2. Geospatial Technologies

Table 6 summarizes five standard geospatial technologies for 3D construction modeling. Geospatial technologies primarily deal with capturing, analyzing, and managing information tied to geographic locations. These data can include things like elevation, land cover, and infrastructure like bridges and highways. While they might involve creating maps or visualizations, the core emphasis is on understanding the spatial relationships and characteristics of geographic features. For remote sensing, data are typically collected via a variety of sensors, including LiDAR, visible-light and infrared cameras, and radar. Remote sensing provides information about land usage, design, and construction in real-time [90]. A previous study [68] proposed a new technique using LiDAR data and multispectral ortho imagery to distinguish between buildings and trees in modeling. The study also used ground height from a digital elevation model (DEM) as a separate source to constitute a ground mask. Another study [91] found that detecting buildings only by height differences may not be sufficient to resolve height ambiguity between buildings and trees. In addition, combining the high accuracy and density of LiDAR point clouds with the visual information from imagery has recently been shown to generate sophisticated and precise 3D building models [32,68,91,92,93,94]. Another study [92] integrated airborne LiDAR data and optical multi-view aerial imagery in two main steps (roof point segmentation and 3D roof model reconstruction), in which a 3D model of a building is reconstructed based on segmentations and 2D lines extracted from optical multi-view aerial photographs. This study contributed a new shrink–expand method (shrinking/reducing LiDAR data size and expanding/enlarging aerial photos).

In addition to GPR, which is an effective survey method that utilizes high-resolution, scalability, and a non-destructive approach [38], GIS, GPS, and RFID are also valuable tools that collect, process, analyze, and visualize geospatial data linked to geographic locations such as coordinates (latitude, longitude), time, and other spatial attributes. For example, previous research [81] developed an integrated framework based on four-dimensional (4D) BIM and GIS modeling to effectively track material logistics and construction activities. The integration of GIS and BIM allows opportunities to facilitate and optimize modeling processes by visualizing, analyzing, and monitoring data. Maps from GPS also help engineers recognize and predict the extent of problems and understand the geographic impact on the far-reaching significance of assets [110,111]. Similarly, global navigation satellite systems (GNSS) and GPS maintain precision, redundancy, and availability of location data, while 3D point clouds, typically generated by data from LiDAR and scanning technologies, may be incorporated into GIS data to create 3D visualization and perform 3D analysis. Another study [104] used GPS data to navigate and support a BIM-AR system for accurate geolocation, spatial reference, scale, and real-time tracking, while a similar study [90] found that geospatial technologies (UAS, LIDAR, and GNSS) play a vital role in the collection, development, storage, management, and dissemination of data for various fields. A case study [90] was conducted on a Utah Department of Transportation (UDOT) highway project that involved the use of small sUAS for monitoring construction progress and GNSS rovers for real-time verification and quantity measurements. The project utilized these technologies to create a 3D model due to the significant geometric complexity of a steep hillside on State Route 20 (SR20). The use of sUAS and GNSS rovers provided numerous benefits, including enhanced productivity of construction inspectors (with a loaded rate of 1.6 times the average hourly rate), increased efficiency through the combined use of technologies such as sUAS, tablets, and GNSS rovers for real-time verification, and a higher confidence score in various cost items.

Finally, RFID technology uses electromagnetic fields to identify and track tags attached to objects. The RFID tags can store information about the 3D models associated with specific construction elements. Any updates made to the 3D models can be linked to the corresponding RFID tags to help track, monitor, and inventory equipment and other assets in various industries, including construction [21].

Researchers [71,105] have also identified technology limitations in the construction applications shown in Table 5. One study [38] found that the complexity of GPR data complicated data interpretation; data processing for GPR requires manual post-processing and user expertise to obtain reliable results. Furthermore, data collection for 3D models is challenging for engineering, especially due to the variety of laws, regulations, and working conditions in specific areas [104,112,113]. Although laser scanning has many benefits, workers must be trained to use and calibrate the laser scanner and process captured data with its various settings, parameters, and software. Lack of proper training can lead to inaccurate and incomplete data collection, resulting in inaccurate or incomplete results. In addition, lasers may be harmful to users’ eyes and skin, so workers must be trained to operate the equipment safely [21,70,114,115]. In terms of legal requirements, some jurisdictions may require individuals and organizations to obtain certifications or licenses to demonstrate competency in the safe and proper use of lasers. A previous study [23] found that the primary challenges to using remote sensing for infrastructure inspection during construction include high costs, a lack of training and relevant skills, a lack of standard contract specifications, and device maintenance and user support.

### 5.3. Algorithm-Based Technologies

Algorithm-based technologies are currently widely used in various industries to facilitate decision-making processes and enhance communication, efficiency, and collaboration. Through the classification method employed in the study, the authors identified algorithm-based technologies. This term emphasizes the considerable reliance on algorithms in the modeling processes within the construction field. Algorithm-based technologies constitute a diverse range of computational tools and methodologies that leverage algorithms as their foundational framework. This classification method stands apart from image technologies, geospatial technology, and automated technologies, highlighting its distinctiveness in the construction context. Algorithm-based technologies such as AR/VR, deep learning, reinforcement learning, and computer vision use algorithms to perform intelligent functions [13,52,57,116,117,118]. AR technology overlays digital information (e.g., images, 3D models, or text) onto the real world, while VR technology immerses users in a completely simulated digital environment. Although AR/VR allows architects and engineers to visualize and present their designs in an immersive environment, deep learning (a subset of AI) reveals multi-part patterns and relationships in data for structural health monitoring, allowing engineers to detect anomalies and predict failures [119,120]. A previous study [36] reconstructed 3D building models using deep learning algorithms and street view imagery with no data from existing building projects.

Computer vision has also effectively improved viewing and management. Computer vision has also become increasingly significant in the construction industry, especially for 3D model viewing and management, because it enables the development of AR experiences in construction. For example, workers can wear AR glasses or use mobile devices to view 3D models overlaid onto a construction site to visualize construction plans and avoid errors [52]. Table 7 presents the three standard algorithm-based technologies and applications for 3D modeling in the construction industry. As shown, AR/VR and deep learning are preferred due to their image and speech recognition capabilities.

However, algorithms trained on historical data may struggle to perform with up-to-date information about the dynamic and intricate nature of the construction process. Furthermore, the algorithms may be obstructed by the inconsistency and lack of comprehensive data on various construction projects, resulting in inaccurate, insufficient, and faulty predictions [52,57]. A previous study [81] identified technical challenges that detrimentally affect construction tasks, but further studies are needed regarding the actual effectiveness of AR/VR for construction projects. One study [116] indicated typical technical limitations of the AI quality inspection model (AI-QIM). Low-depth image resolution of 1280 by 720 pixels does not permit precise detection at distances greater than 2 m from the viewer, and it requires a significant amount of time for training and considerable computational resources [81,82]. Depending on the model complexity, dataset size, and available computational resources, training for deep learning models, such as neural networks, may take a long time.

The continued development of algorithm-based technology may result in more innovative and groundbreaking applications for the construction industry. For example, algorithms may be programmed to make predictions based on large amounts of data, such as material costs or labor availabilities, resulting in more informed construction decisions. Mobile and web-based construction software also connects construction teams in the field during reporting, plan viewing, punch list completion, and scheduling [110,134,135,136]. For example, a previous study [136] developed a web-based 3D visualization framework using open-source technologies to collect oceanic information, providing increased rendering speed, high visual effects, and on-the-fly 3D visualization of oceanographic data. Therefore, future developments should focus on mobile web-based construction software that increases accessibility and usability and enhances interactivity and animation, which are essential in the digital age.

### 5.4. Automated Technologies

Automated technologies such as robots, UAVs, and UAS have increased workplace safety by substituting for humans in hazardous and high-risk environments. A notable increase in attention and resources dedicated to the application and operation of these technologies has occurred over the past decade because automation ensures high accuracy and consistency in manufacturing and data collection [9,124,130,137]. Robots offer several advantages over traditional manual labor, including increased productivity, reduced costs, and improved safety. One study [86] integrated images from a single camera mounted on a mobile robot for 3D modeling, utilizing a volumetric model built in real-time for obstacle detection while the surface model was developed offline. UAVs/UASs, also known as drones, are increasingly employed in construction for inspection, surveying, and safety monitoring. A previous study [46] combined UAVs and BIM to develop a genetic algorithm and confirm that UAVs are now used widely, especially for unreachable areas. In the study, BIM provided critical information, such as the distance between adjacent viewpoints, longitude and latitude, yaw angle, and flight direction of the initial view. The path optimization for UAV/UAS inspections (safe distance of UAV/UAS inspection, target areas, and centroid projection) made the inspection process of UAVs/UASs automatic. These intelligent methodologies provide precise information to build surface images, thereby improving automation in the construction industry. Table 8 lists automated technologies for 3D modeling of construction projects.

However, researchers have identified several limitations of automated technologies for construction projects (Table 5). For example, UAVs cannot work in inclement weather conditions (e.g., strong wind, rain) [46,105,112,138], and in some remote areas, UAVs must operate manually. In addition, robotic inspection systems require considerable costs for purchase, installation, training, and data processing [9,139,140,141,142]. Robots may also struggle in unexpected situations to interpret and comprehend the broad context of a construction project, such as timelines, construction plans, and customer requirements. Future studies should address these limitations to enhance the adaptability and flexibility of automated technologies.

**Table 8:** Automated technologies for 3D models.

No.	Technology	Description and Typical Application	Key References	Number of References
1	Robots	The utilization of robots in construction is a growing trend that promises to revolutionize the field. Robots may be used to survey and map construction sites, increasing understanding of project progress and obstacles.	[6,9,86,114,130,137,140,141]	8
2	UAVs/UASs	UAVs are used widely in a range of industries for 3D modeling, including surveying, mapping, construction, and environmental monitoring, because of their advantages (e.g., reduced cost, increased safety, and high accuracy).UASs, commonly referred to as unmanned aerial systems, unmanned aerial vehicles, remotely piloted aircraft systems, and drones, benefit from 3D modeling processes. They may assess pre-project and project survey data, site inspection, surveillance, tracking, and management.	[33,46,109,143,144,145,146,147]	8

### 5.5. Software for 3D Modeling

Various software platforms have recently emerged for 3D modeling, such as Bentley SYNCHRO, Unreal Engine 5 (UE5), Autodesk Construction Cloud (ACC), Quadri, and Trimble Business Center, as shown in Table 9. Bentley SYNCHRO and Quadri are 4D construction modeling and scheduling software that combine 3D models with project schedules to generate an integrated visual representation of a construction project. Using Bentley SYNCHRO, construction engineers may link BIM with project schedules to visualize the progression of a construction project. Engineers can present the construction plan and schedule, enabling stakeholder collaboration and commitment with confidence, leading to on-time execution and delivery, reduced risk, and quicker approvals of payments. Web and mobile applications of these software products extend the value of the 4D construction model by exchanging and managing 2D, 3D, and 4D models in full project context, including time, location, and task priority. UE5 is an effective tool for creating materials and virtual worlds, sculpting landscapes, and painting foliage. Currently, UE5 introduces resources such as Auto-Landscape Master Material and the UE5 Planet Tutorial. Auto-Landscape Master Material creates and manages materials for landscapes within the engine. The ACC includes mobile apps and field management tools that enable on-site construction teams to access project information, capture field data, and collaborate with other partners in real-time. ACC is more appropriate for construction sites than traditional Autodesk which is primarily desktop-based and limited to field use.

Pix4Dcloud is photogrammetry software that allows users to generate a project for online processing and share their reconstruction with other stakeholders. Engineers may use Pix4Dcloud to upload images to obtain accurate, ready-to-use 2D maps and 3D models, access data for up-to-date project records, and rapidly estimate the volume of stockpiles in quarries or buildings. Pix4Dcloud is also a valuable tool to save time and improve accuracy by automatically marking ground control points, verifying construction site progress by comparing the as-built to the design, initially determining errors, and avoiding costly rework. The software helps ensure smooth collaboration and accelerate productivity by exporting and sharing data with stakeholders. Table 9 describes the advantages and disadvantages of this 3D modeling software.

## 6. Conclusions

This research provides a systematic and comprehensive review of the use of various technologies for 3D models in the construction industry. The findings from this study indicate that BIM is the predominant tool for 3D modeling, but BIM limitations, such as complicated implementation and different standardizations, legal and contractual issues, complicated training and skill development, and extensive data management, should be considered.

This study provides four main contributions to the body of knowledge and the construction industry. First, the study highlighted the potential capacities of 3D models to support construction design, combined with hybrid approaches to generate accurate models while manipulating and adjusting changes for customer demand. Second, because BIM is increasingly prevalent and variable across firms and AEC practices, meaning its standardizations and workflows may change considerably, an investigation of BIM applications and standards in various locations and laws is vitally necessary. Third, the authors summarized prominent future research directions for 3D models based on the observed advantages and limitations. Fourth, the systematic review of AI in this study revealed the need for an in-depth study of AI in construction because it may accelerate the process of generating models, monitoring equipment in real-time, predicting maintenance, and optimizing project planning.

In future studies, researchers should integrate two or more technologies, such as construction inspection using robotic technologies and BIM, BIM-AR systems, mobile and web-based construction software, and algorithm-based technologies. The integration of BIM and AI is especially promising and can potentially transform the construction industry. Developing an AI-BIM system may facilitate automated quality control and construction processes, mitigating errors and inconsistencies, forecasting maintenance demands, and ensuring long-term sustainability. Exploring the combination and utilization of BIM, AI, and automation for the design and construction activities in innovative 3D models is also a promising direction.

## Figures and Tables

**Table 1:** Profile of articles for 3D models.

No.	Journals	Number of Articles		Proportions of Analyzed Articles	CiteScore	Impact Factor
		Selected	Analyzed			
1	Automation in Construction	74	29	32.2	15.0	10.50
2	International Journal of Photogrammetry and Remote Sensing	33	16	17.8	17.6	11.77
3	Journal of Computing in Civil Engineering (JCCE)	26	9	10.0	7.6	10.00
4	Journal of Construction Engineering and Management (JCEM)	15	8	8.9	6.4	6.20
5	Advanced Engineering Informatics (AEI)	15	5	5.6	10.1	7.86
6	Tunneling and Underground Space Technology (TUST)	4	2	2.2	11.0	6.41
7	Construction and Building Materials (CBM)	4	1	1.1	10.6	7.69
8	Robotics and Autonomous Systems (RAS)	4	4	4.4	8.1	3.70
9	Journal of Building Engineering (JOBE)	4	2	2.2	5.5	5.60
10	Digital Applications in Archaeology and Cultural Heritage (DAACH)	3	1	1.1	5.0	3.47
11	Journal of Bridge Engineering (JBE)	3	1	1.1	6.4	7.14
12	International Journal of Geomechanics (IJG)	3	1	1.1	6.3	3.93
13	Journal of Management in Engineering (JME)	3	2	2.2	7.9	9.00
14	Computer-Aided Design (CAD)	3	1	1.1	6.1	3.65
15	Journal of Aerospace Engineering (JAE)	2	2	2.2	3.5	2.24
16	Image and Vision Computing (IVC)	2	1	1.1	6.3	3.86
17	International Journal of Applied Earth Observation and Geoinformation (IJAEOG)	2	1	1.1	10.5	7.67
18	Journal of Cultural Heritage (JCH)	2	1	1.1	6.1	3.23
19	Computers in Industry (CI)	1	1	1.1	16.9	11.25
20	Computers & Geosciences (CG)	1	1	1.1	7.0	5.17
21	Journal of Industrial Information Integration (JIII)	1	1	1.1	20.9	11.72
Total		205	90	100		

**Table 2:** Technologies used for 3D modeling.

No.	Technology	Frequency	Percentage of Identified Technologies (%)
1	BIM	77	20.8
2	Remote sensing	71	19.1
3	Stereo vision system/Photo processing programs	57	15.4
4	Augmented reality (AR)/Virtual reality (VR)	34	9.2
5	Deep learning, Reinforcement learning	23	7.3
6	Robots	16	4.3
7	Unmanned aerial vehicles (UAV)/Unmanned aircraft systems (UAS)	15	4.0
8	Topological model	9	2.4
9	Mesh simplification	9	2.4
10	Geographic information system (GIS)/Global positioning system (GPS)	8	2.2
11	Computer Vision	6	1.6
12	Finite element method	5	1.3
13	Web-based applications	5	1.3
14	Machine learning	4	1.1
15	Extended reality (ER)	3	0.8
16	Discrete element method	2	0.5
17	Others	27	7.3
Total		371	100.0

**Table 3:** Modeled elements in 3D models.

No.	Modeled Element	Description	Key References	Frequency (*n* = 205)	Percentage of Nodes (%)
1	Buildings	Three-dimensional models may illustrate a series of building sections: foundation, wall, rooftop, electricity system, ventilation system, room facilities	[26,30,31]	134	48.0
2	Street view, earth surface, and building blocks	Three-dimensional models can be used to model streets, tree segmentations, building blocks, and surfaces of unstructured areas	[32,33]	45	16.1
3	Urban planning (e.g., city and cultural heritage)	Three-dimensional models can include 3D city models and cultural heritage for tourism and conservation purposes	[34,35]	34	12.2
4	Infrastructure (e.g., tunnel, bridge, road, and dam)	Three-dimensional models may visualize, optimize, and validate for dams, tunnels, roads, bridge designs, and operations	[17]	34	12.2
5	Activity in construction	Three-dimensional building models are an established instance of geospatial information in built environments	[36,37]	21	7.5
6	Building materials (e.g., aggregate and sand)	Three-dimensional models are effective tools for modeling and controlling the material quality	[38,39]	7	2.5
7	Room facilities (e.g., chairs and tables)	Three-dimensional models can be used to visualize and enhance mesh construction for room facilities	[40]	3	1.1
8	Transportation operation	Three-dimensional models are used to visualize transport operations in construction	[41]	1	0.4
Total				279	100.0

**Table 5:** Advantages and limitations of 3D modeling technologies.

No.	Technologies	Advantages	Limitations	Typical References
1	BIM	Covers various mechanical, electrical, and plumbing components Extracts accurate component module Clash detection May create an as-built model for facility management	Heavily relies on the excellent coverage of collected data Lacks sufficient legal framework	[24,80]
2	Stereo vision system/Photo processing programs	May capture depth information Less affected by light Provides immediate processing and response	Requires exact alignment Requires a complex computational processing	[68]
3	GIS/GPS	Streamlines the management of large blocks Improves interpretation of the inspection outcome Facilitates BIM applications Presents all types of geographical data	Loss of data during transformation Minimal level of detail harmonization during the mapping process Transformation of the local placement system to a world coordinate system	[81]
4	AR/VR	AR may produce safer working environments VR may support safety education Uses for life cycle assessment	Lack of awareness of the technology Poor usability Significant time commitment for implementation Lack of interoperability between BIM systems and AR/VR models	[82,83]
5	Deep learning, Reinforcement learning	High accuracy Automatically learns features from raw data May scale to handle large datasets	Requires large amounts of training data Requires significant computational resources Some models are difficult to interpret	[84,85,86]
6	Robot	Improves safety in inspection, survey Works faster and more consistently Reduces labor costs for investigating remote areas No need to suspend traffic during the cable inspection robot	Requires a high initial cost Requires regular maintenance and repair Introduces new safety concerns	[9,87]
7	Remote sensing	May generate an accurate spatial representation of buildings quickly	Time-consuming in some cases Provides point clouds (unstructured data)	[62,88]

**Table 6:** Geospatial technologies for 3D models.

No.	Technology	Description and Typical Application	Key References	Number of References
1	Remote sensing	Remote sensing data are typically collected using a variety of sensors, including visible and infrared cameras, radar, and light detecting and ranging. Remote sensing provides information about land usage, the earth’s surface, and infrastructure.	[32,68,84,87,95,96,97,98,99]	9
2	GPR	Benefits of using GPR for 3D modeling include improved site understanding, design, and construction in real-time. GPR data allow proper planning to avoid accidental damage to utilities during excavation.	[38,100,101,102]	4
3	GIS	GIS enhances visualization, improves decision-making and flexibility, and increases efficiency, scalability, and accessibility. Users may manipulate and analyze spatial data and create maps.	[63,81,103,104]	4
4	GPS	GPS is a satellite-based navigation system that provides location and time information to users worldwide. It beneficially allows accurate geolocation, spatial reference and scale, real-time tracking, and monitoring.	[105,106,107]	3
5	RFID	Using RFID in conjunction with computer-aided design (CAD), BIM, and other forms (GIS, GPS, and GPR) of 3D modeling may also strengthen jobsite management by reducing errors and rework.	[21,108,109]	3

**Table 7:** Algorithm-based technologies for 3D models.

No.	Technology	Description and Typical Application	Key References	Number of References
1	AR/VR	AR/VR has demonstrated powerful capabilities across multiple industries, including entertainment, manufacturing, and construction.	[14,19,41,50,81,117,121,122,123,124]	10
2	Deep learning, reinforcement learning	Deep learning is a subfield of machine learning that applies neural networks to recognize complex patterns and relationships in data for structural health monitoring, allowing users to detect anomalies and predict failures. It has become increasingly popular because of its capabilities in image and speech recognition and autonomous tools.	[36,65,119,125,126,127,128,129,130]	9
3	Computer vision	Computer vision has a series of applications in the construction field, including construction equipment detection, automated inspection with 3D scanning of infrastructures, and detection of structural defects in buildings.	[52,131,132,133]	4

**Table 9:** Software for 3D models.

No.	Software	Advantage	Disadvantage	Reference
1	Bentley SYNCHRO (v6.4)	May model BIM 4D planning Allows users to share project data and collaborate on simulations Provides a variety of reporting tools	Requires excessive storage space Time must be proficient Potentially difficult data transitioning	[148]
2	Quadri (version 2021)	Compatible with SketchUp, Revit, Tekla structure, Quantum Cloud-based software Has a user-friendly interface	May require time to become proficient May be complex due to the vast amount of data like BIM	[149]
3	Unreal Engine 5 (version 5)	Real-time rendering; logistics animations; make changes in real-time Improve workflows and productivity	Difficult to customize Potential bugs or performance issues	[150]
4	Autodesk Construction Cloud (2021 version of Revit)	Easy to collaborate on projects from anywhere Strong security and compliance features	Features not fully developed Potentially expensive, especially for large projects	[13]
5	Pix4Dcloud (2021 version)	Offers an array of tools: viewing, managing, and analyzing reality May create a project for online processing	Subscription-based software Potentially expensive for users	[151]

## Data Availability

Data are available upon request from the authors.

## References

[1] Pan Z., Yu Y., Xiao F., Zhang J. (2023). Recovering Building Information Model from 2D Drawings for Mechanical, Electrical and Plumbing Systems of Ageing Buildings. Autom. Constr..

[2] Zhao Y., Deng X., Lai H. (2021). Reconstructing BIM from 2D Structural Drawings for Existing Buildings. Autom. Constr..

[3] Wen R., Tang W., Su Z. (2017). Topology Based 2D Engineering Drawing and 3D Model Matching for Process Plant. Graph. Models.

[4] He Y., Yang Y., He T., Lai Y., He Y., Chen B. (2024). Small and Micro-Water Quality Monitoring Based on the Integration of a Full-Space Real 3D Model and IoT. Sensors.

[5] van Manen M., olde Scholtenhuis L., Voordijk H. (2022). Empirically Validating Five Propositions Regarding 3D Visualizations for Subsurface Utility Projects. Eng. Constr. Archit. Manag..

[6] Lin J.J., Ibrahim A., Sarwade S., Golparvar-Fard M. (2021). Bridge Inspection with Aerial Robots: Automating the Entire Pipeline of Visual Data Capture, 3D Mapping, Defect Detection, Analysis, and Reporting. J. Comput. Civ. Eng..

[7] Mohamed M., Tran D.Q. (2022). Content Analysis of E-Inspection Implementation for Highway Infrastructure Construction Projects. Eng. Constr. Arch. Manag..

[8] Mohamed M., Tran D.Q. (2021). Risk-Based Inspection for Concrete Pavement Construction Using Fuzzy Sets and Bayesian Networks. Autom. Constr..

[9] Hou S., Dong B., Wang H., Wu G. (2020). Inspection of Surface Defects on Stay Cables Using a Robot and Transfer Learning. Autom. Constr..

[10] Alavi H., Bortolini R., Forcada N. (2022). BIM-Based Decision Support for Building Condition Assessment. Autom. Constr..

[11] Garcia-Gago J., Sánchez-Aparicio L.J., Soilán M., González-Aguilera D. (2022). HBIM for Supporting the Diagnosis of Historical Buildings: Case Study of the Master Gate of San Francisco in Portugal. Autom. Constr..

[12] Resende M.M., Gambare E.B., Silva L.A., Cordeiro Y.D.S., Almeida E., Salvador R.P. (2022). Infrared Thermal Imaging to Inspect Pathologies on Façades of Historical Buildings: A Case Study on the Municipal Market of São Paulo, Brazil. Case Stud. Constr. Mater..

[13] Abdirad H., Mathur P. (2021). Artificial Intelligence for BIM Content Management and Delivery: Case Study of Association Rule Mining for Construction Detailing. Adv. Eng. Inform..

[14] Chen H., Hou L., Zhang G.K., Moon S. (2021). Development of BIM, IoT and AR/VR Technologies for Fire Safety and Upskilling. Autom. Constr..

[15] Park J., Cai H., Dunston P.S., Ghasemkhani H. (2017). Database-Supported and Web-Based Visualization for Daily 4D BIM. J. Constr. Eng. Manag..

[16] Siebelink S., Voordijk J.T., Adriaanse A. (2018). Developing and Testing a Tool to Evaluate BIM Maturity: Sectoral Analysis in the Dutch Construction Industry. J. Constr. Eng. Manag..

[17] Vignali V., Acerra E.M., Lantieri C., Di Vincenzo F., Piacentini G., Pancaldi S. (2021). Building Information Modelling (BIM) Application for an Existing Road Infrastructure. Autom. Constr..

[18] Alirezaei S., Taghaddos H., Ghorab K., Tak A.N., Alirezaei S. (2022). BIM-Augmented Reality Integrated Approach to Risk Management. Autom. Constr..

[19] Panya D.S., Kim T., Choo S. (2023). An Interactive Design Change Methodology Using a BIM-Based Virtual Reality and Augmented Reality. J. Build. Eng..

[20] Dino I.G., Sari A.E., Iseri O.K., Akin S., Kalfaoglu E., Erdogan B., Kalkan S., Alatan A.A. (2020). Image-Based Construction of Building Energy Models Using Computer Vision. Autom. Constr..

[21] Valero E., Adán A., Bosché F. (2016). Semantic 3D Reconstruction of Furnished Interiors Using Laser Scanning and RFID Technology. J. Comput. Civ. Eng..

[22] Jiang Y., Yang G., Li H., Zhang T. (2023). Knowledge Driven Approach for Smart Bridge Maintenance Using Big Data Mining. Autom. Constr..

[23] Tran D., Harper C., Sturgill R., National Cooperative Highway Research Program, Transportation Research Board (2022). National Academies of Sciences, Engineering, and Medicine. Highway Infrastructure Inspection Practices for the Digital Age.

[24] Lee S., Yu J., Jeong D. (2015). BIM Acceptance Model in Construction Organizations. J. Manag. Eng..

[25] Benjaoran V., Bhokha S. (2009). Enhancing Visualization of 4D CAD Model Compared to Conventional Methods. Eng. Constr. Archit. Manag..

[26] Chen Y.-J., Lai Y.-S., Lin Y.-H. (2020). BIM-Based Augmented Reality Inspection and Maintenance of Fire Safety Equipment. Autom. Constr..

[27] Taylor T., Sturgill R., Waddle S., Li Y., Goodrum P., Molenaar K., Al-Haddad S. (2020). Workforce Optimization Workbook for Transportation Construction Projects.

[28] Siraj N.B., Fayek A.R. (2019). Risk Identification and Common Risks in Construction: Literature Review and Content Analysis. J. Constr. Eng. Manag..

[29] Kazmi H., Fu C., Miller C. (2023). Ten Questions Concerning Data-Driven Modelling and Forecasting of Operational Energy Demand at Building and Urban Scale. Build. Environ..

[30] Martínez-Rocamora A., García-Alvarado R., Casanova-Medina E., González-Böhme L.F., Auat-Cheein F. (2020). Parametric Programming of 3D Printed Curved Walls for Cost-Efficient Building Design. J. Constr. Eng. Manag..

[31] Nepal M.P., Staub-French S., Pottinger R., Zhang J. (2013). Ontology-Based Feature Modeling for Construction Information Extraction from a Building Information Model. J. Comput. Civ. Eng..

[32] Fekete A., Cserep M. (2021). Tree Segmentation and Change Detection of Large Urban Areas Based on Airborne LiDAR. Comput. Geosci..

[33] Zheng X., Wang F., Li Z. (2018). A Multi-UAV Cooperative Route Planning Methodology for 3D Fine-Resolution Building Model Reconstruction. ISPRS J. Photogramm. Remote Sens..

[34] Bosché F. (2012). Plane-Based Registration of Construction Laser Scans with 3D/4D Building Models. Adv. Eng. Inform..

[35] Chen K., Lu W., Xue F., Tang P., Li L.H. (2018). Automatic Building Information Model Reconstruction in High-Density Urban Areas: Augmenting Multi-Source Data with Architectural Knowledge. Autom. Constr..

[36] Pang H.E., Biljecki F. (2022). 3D Building Reconstruction from Single Street View Images Using Deep Learning. Int. J. Appl. Earth Obs. Geoinf..

[37] Xu Y., Shen X., Lim S. (2021). CorDet: Corner-Aware 3D Object Detection Networks for Automated Scan-to-BIM. J. Comput. Civ. Eng..

[38] Lombardi F., Lualdi M., Garavaglia E. (2021). Masonry Texture Reconstruction for Building Seismic Assessment: Practical Evaluation and Potentials of Ground Penetrating Radar Methodology. Constr. Build. Mater..

[39] Xiao Y., Tutumluer E. (2017). Gradation and Packing Characteristics Affecting Stability of Granular Materials: Aggregate Imaging-Based Discrete Element Modeling Approach. Int. J. Geomech..

[40] Fahim G., Amin K., Zarif S. (2022). Enhancing Single-View 3D Mesh Reconstruction with the Aid of Implicit Surface Learning. Image Vis. Comput..

[41] Chen H.-M., Huang P.-H. (2013). 3D AR-Based Modeling for Discrete-Event Simulation of Transport Operations in Construction. Autom. Constr..

[42] Mirzaei A., Nasirzadeh F., Parchami Jalal M., Zamani Y. (2018). 4D-BIM Dynamic Time–Space Conflict Detection and Quantification System for Building Construction Projects. J. Constr. Eng. Manag..

[43] Pikas E., Sacks R., Hazzan O. (2013). Building Information Modeling Education for Construction Engineering and Management. II: Procedures and Implementation Case Study. J. Constr. Eng. Manag..

[44] Gharaibeh L., Matarneh S., Eriksson K., Lantz B. (2023). Digital Transformation of the Wood Construction Supply Chain through Building Information Modelling: Current State of Practice. Constr. Innov..

[45] Ullah K., Lill I., Witt E., Lill I., Witt E. (2019). An overview of BIM adoption in the construction industry: Benefits and barriers. Emerald Reach Proceedings Series.

[46] Tan Y., Li S., Liu H., Chen P., Zhou Z. (2021). Automatic Inspection Data Collection of Building Surface Based on BIM and UAV. Autom. Constr..

[47] Tang S., Li X., Zheng X., Wu B., Wang W., Zhang Y. (2022). BIM Generation from 3D Point Clouds by Combining 3D Deep Learning and Improved Morphological Approach. Autom. Constr..

[48] Tsai Y.-H., Wang J., Chien W.-T., Wei C.-Y., Wang X., Hsieh S.-H. (2019). A BIM-Based Approach for Predicting Corrosion under Insulation. Autom. Constr..

[49] Inzerillo L., Di Mino G., Roberts R. (2018). Image-Based 3D Reconstruction Using Traditional and UAV Datasets for Analysis of Road Pavement Distress. Autom. Constr..

[50] Zhao S., Kang F., Li J., Ma C. (2021). Structural Health Monitoring and Inspection of Dams Based on UAV Photogrammetry with Image 3D Reconstruction. Autom. Constr..

[51] Chen S.-H., Xue F. (2023). Automatic BIM Detailing Using Deep Features of 3D Views. Autom. Constr..

[52] Huang M.Q., Ninić J., Zhang Q.B. (2021). BIM, Machine Learning and Computer Vision Techniques in Underground Construction: Current Status and Future Perspectives. Tunn. Undergr. Space Technol..

[53] Zhang S., Teizer J., Lee J.-K., Eastman C.M., Venugopal M. (2013). Building Information Modeling (BIM) and Safety: Automatic Safety Checking of Construction Models and Schedules. Autom. Constr..

[54] Anane W., Iordanova I., Ouellet-Plamondon C. (2023). BIM-Driven Computational Design for Robotic Manufacturing in off-Site Construction: An Integrated Design-to-Manufacturing (DtM) Approach. Autom. Constr..

[55] Bynum P., Issa R.R.A., Olbina S. (2013). Building Information Modeling in Support of Sustainable Design and Construction. J. Constr. Eng. Manag..

[56] Chen J., Lu W., Fu Y., Dong Z. (2023). Automated Facility Inspection Using Robotics and BIM: A Knowledge-Driven Approach. Adv. Eng. Inform..

[57] Demirdöğen G., Işık Z., Arayici Y. (2023). BIM-Based Big Data Analytic System for Healthcare Facility Management. J. Build. Eng..

[58] Herrera R.F., Mourgues C., Alarcón L.F., Pellicer E. (2021). Analyzing the Association between Lean Design Management Practices and BIM Uses in the Design of Construction Projects. J. Constr. Eng. Manag..

[59] Grifoni E., Legnaioli S., Nieri P., Campanella B., Lorenzetti G., Pagnotta S., Poggialini F., Palleschi V. (2018). Construction and Comparison of 3D Multi-Source Multi-Band Models for Cultural Heritage Applications. J. Cult. Herit..

[60] Hosseininaveh Ahmadabadian A., Karami A., Yazdan R. (2019). An Automatic 3D Reconstruction System for Texture-Less Objects. Robot. Auton. Syst..

[61] Hu F., Wang F., Ren Y., Xu F., Qiu X., Ding C., Jin Y. (2022). Error Analysis and 3D Reconstruction Using Airborne Array InSAR Images. ISPRS J. Photogramm. Remote Sens..

[62] Sung C., Kim P.Y. (2016). 3D Terrain Reconstruction of Construction Sites Using a Stereo Camera. Autom. Constr..

[63] Suveg I., Vosselman G. (2004). Reconstruction of 3D Building Models from Aerial Images and Maps. ISPRS J. Photogramm. Remote Sens..

[64] Elhashash M., Qin R. (2022). Cross-View SLAM Solver: Global Pose Estimation of Monocular Ground-Level Video Frames for 3D Reconstruction Using a Reference 3D Model from Satellite Images. ISPRS J. Photogramm. Remote Sens..

[65] Gao J., Liu J., Ji S. (2023). A General Deep Learning Based Framework for 3D Reconstruction from Multi-View Stereo Satellite Images. ISPRS J. Photogramm. Remote Sens..

[66] Nettis A., Massimi V., Nutricato R., Nitti D.O., Samarelli S., Uva G. (2023). Satellite-Based Interferometry for Monitoring Structural Deformations of Bridge Portfolios. Autom. Constr..

[67] Stucker C., Schindler K. (2022). ResDepth: A Deep Residual Prior for 3D Reconstruction from High-Resolution Satellite Images. ISPRS J. Photogramm. Remote Sens..

[68] Awrangjeb M., Zhang C., Fraser C.S. (2013). Automatic Extraction of Building Roofs Using LIDAR Data and Multispectral Imagery. ISPRS J. Photogramm. Remote Sens..

[69] Golparvar-Fard M., Peña-Mora F., Savarese S. (2015). Automated Progress Monitoring Using Unordered Daily Construction Photographs and IFC-Based Building Information Models. J. Comput. Civ. Eng..

[70] Hu Y., Chen X., Tang Z., Yu J., Chen Y., Wu Z., Yang D., Chen Y. (2021). Collaborative 3D Real Modeling by Multi-View Images Photogrammetry and Laser Scanning: The Case Study of Tangwei Village, China. Digit. Appl. Archaeol. Cult. Herit..

[71] Kingsland K. (2020). Comparative Analysis of Digital Photogrammetry Software for Cultural Heritage. Digit. Appl. Archaeol. Cult. Herit..

[72] Zheng Q., Shi B., Pan G. (2020). Summary Study of Data-Driven Photometric Stereo Methods. Virtual Real. Intell. Hardw..

[73] Shi B., Mo Z., Wu Z., Duan D., Yeung S.-K., Tan P. (2019). A Benchmark Dataset and Evaluation for Non-Lambertian and Uncalibrated Photometric Stereo. IEEE Trans. Pattern Anal. Mach. Intell..

[74] Ju Y., Lam K.-M., Xie W., Zhou H., Dong J., Shi B. (2024). Deep Learning Methods for Calibrated Photometric Stereo and Beyond. IEEE Trans. Pattern Anal. Mach. Intell..

[75] Horna S., Meneveaux D., Damiand G., Bertrand Y. (2009). Consistency Constraints and 3D Building Reconstruction. Comput. Aided Des..

[76] Khalili A., Chua D.K.H. (2015). IFC-Based Graph Data Model for Topological Queries on Building Elements. J. Comput. Civ. Eng..

[77] Yang S., Cai G., Du J., Chen P., Su J., Wu Y., Wang Z., Li J. (2022). Connectivity-Aware Graph: A Planar Topology for 3D Building Surface Reconstruction. ISPRS J. Photogramm. Remote Sens..

[78] Li M., Nan L. (2021). Feature-Preserving 3D Mesh Simplification for Urban Buildings. ISPRS J. Photogramm. Remote Sens..

[79] Zhang S., Zhan Y., Cui X., Gao M., Huang J., Metaxas D. (2013). 3D Anatomical Shape Atlas Construction Using Mesh Quality Preserved Deformable Models. Comput. Vis. Image Underst..

[80] Wang B., Wang Q., Cheng J.C.P., Song C., Yin C. (2022). Vision-Assisted BIM Reconstruction from 3D LiDAR Point Clouds for MEP Scenes. Autom. Constr..

[81] Deng Y., Cheng J.C.P., Anumba C. (2016). Mapping between BIM and 3D GIS in Different Levels of Detail Using Schema Mediation and Instance Comparison. Autom. Constr..

[82] Davila Delgado J.M., Oyedele L., Demian P., Beach T. (2020). A Research Agenda for Augmented and Virtual Reality in Architecture, Engineering and Construction. Adv. Eng. Inform..

[83] Osorto Carrasco M.D., Chen P.-H. (2021). Application of Mixed Reality for Improving Architectural Design Comprehension Effectiveness. Autom. Constr..

[84] Yin Y., Antonio J. (2020). Application of 3D Laser Scanning Technology for Image Data Processing in the Protection of Ancient Building Sites through Deep Learning. Image Vis. Comput..

[85] Yu D., Ji S., Liu J., Wei S. (2021). Automatic 3D Building Reconstruction from Multi-View Aerial Images with Deep Learning. ISPRS J. Photogramm. Remote Sens..

[86] Trzeciak M., Brilakis I. (2023). Dense 3D Reconstruction of Building Scenes by AI-Based Camera–Lidar Fusion and Odometry. J. Comput. Civ. Eng..

[87] Einhorn E., Schröter C., Gross H.M. (2011). Attention-Driven Monocular Scene Reconstruction for Obstacle Detection, Robot Navigation and Map Building. Robot. Auton. Syst..

[88] Nikoohemat S., Diakité A.A., Zlatanova S., Vosselman G. (2020). Indoor 3D Reconstruction from Point Clouds for Optimal Routing in Complex Buildings to Support Disaster Management. Autom. Constr..

[89] Tian Y., Gerke M., Vosselman G., Zhu Q. (2010). Knowledge-Based Building Reconstruction from Terrestrial Video Sequences. ISPRS J. Photogramm. Remote Sens..

[90] Mallela J., Mitchell A., Gustafson J., Olsen M.J., Parrish C., Gillins D.T., Kumpula M., Roe G. (2018). Effective Use of Geospatial Tools in Highway Construction.

[91] Jung J., Sohn G. (2019). A Line-Based Progressive Refinement of 3D Rooftop Models Using Airborne LiDAR Data with Single View Imagery. ISPRS J. Photogramm. Remote Sens..

[92] Cheng L., Tong L., Chen Y., Zhang W., Shan J., Liu Y., Li M. (2013). Integration of LiDAR Data and Optical Multi-View Images for 3D Reconstruction of Building Roofs. Opt. Lasers Eng..

[93] Wu Q., Yang H., Wei M., Remil O., Wang B., Wang J. (2018). Automatic 3D Reconstruction of Electrical Substation Scene from LiDAR Point Cloud. ISPRS J. Photogramm. Remote Sens..

[94] Catharia O., Richard F., Vignoles H., Véron P., Aoussat A., Segonds F. (2023). Smartphone LiDAR Data: A Case Study for Numerisation of Indoor Buildings in Railway Stations. Sensors.

[95] Asadi K., Haritsa V.R., Han K., Ore J.-P. (2021). Automated Object Manipulation Using Vision-Based Mobile Robotic System for Construction Applications. J. Comput. Civ. Eng..

[96] Carter-Greaves L.E., Eyre M., Vogt D., Coggan J. (2023). Algorithm Development for Automated Key Block Analysis in Tunnels from LiDAR Point Cloud Data. Tunn. Undergr. Space Technol..

[97] Huang H., Brenner C., Sester M. (2013). A Generative Statistical Approach to Automatic 3D Building Roof Reconstruction from Laser Scanning Data. ISPRS J. Photogramm. Remote Sens..

[98] Cheng L., Yuan Y., Xia N., Chen S., Chen Y., Yang K., Ma L., Li M. (2018). Crowd-Sourced Pictures Geo-Localization Method Based on Street View Images and 3D Reconstruction. ISPRS J. Photogramm. Remote Sens.

[99] Avdan U., Kaplan G., Küçük Matcı D., Yiğit Avdan Z., Erdem F., Tuğba Mızık E., Demirtaş İ. (2022). Soil Salinity Prediction Models Constructed by Different Remote Sensors. Phys. Chem. Earth Parts A/B/C.

[100] Ahmed H., La H.M., Tran K. (2020). Rebar Detection and Localization for Bridge Deck Inspection and Evaluation Using Deep Residual Networks. Autom. Constr..

[101] Calhoon T., Zegeye E., Velasquez R., Calvert J. (2022). Using Falling Weight Deflectometer (FWD) and Ground Penetrating Radar (GPR) to Monitor the Effects of Seasonal Moisture Variation on the Structural Capacity of Pavements. Constr. Build. Mater..

[102] Khamzin A.K., Varnavina A.V., Torgashov E.V., Anderson N.L., Sneed L.H. (2017). Utilization of Air-Launched Ground Penetrating Radar (GPR) for Pavement Condition Assessment. Constr. Build. Mater..

[103] Deng Y., Gan V.J.L., Das M., Cheng J.C.P., Anumba C. (2019). Integrating 4D BIM and GIS for Construction Supply Chain Management. J. Constr. Eng. Manag..

[104] Sánchez-Aparicio L.J., Masciotta M.-G., García-Alvarez J., Ramos L.F., Oliveira D.V., Martín-Jiménez J.A., González-Aguilera D., Monteiro P. (2020). Web-GIS Approach to Preventive Conservation of Heritage Buildings. Autom. Constr..

[105] Garbett J., Hartley T., Heesom D. (2021). A Multi-User Collaborative BIM-AR System to Support Design and Construction. Autom. Constr..

[106] Cai H., Andoh A.R., Su X., Li S. (2014). A Boundary Condition Based Algorithm for Locating Construction Site Objects Using RFID and GPS. Adv. Eng. Inform.

[107] Strach M., Dronszczyk P. (2016). Comprehensive 3D Measurements of Tram Tracks in the Tunnel Using the Combination of Laser Scanning Technology and Traditional TPS/GPS Surveying. Transp. Res. Procedia.

[108] Domdouzis K., Kumar B., Anumba C. (2007). Radio-Frequency Identification (RFID) Applications: A Brief Introduction. Adv. Eng. Inform..

[109] Zhang Y., Bai L. (2015). Rapid Structural Condition Assessment Using Radio Frequency Identification (RFID) Based Wireless Strain Sensor. Autom. Constr..

[110] Park J., Im S., Lee K.-H., Lee J.-O. (2012). Vision-Based SLAM System for Small UAVs in GPS-Denied Environments. J. Aerosp. Eng..

[111] Zhang S., Hou D., Wang C., Pan F., Yan L. (2020). Integrating and Managing BIM in 3D Web-Based GIS for Hydraulic and Hydropower Engineering Projects. Autom. Constr..

[112] Jiang W., Zhou Y., Ding L., Zhou C., Ning X. (2020). UAV-Based 3D Reconstruction for Hoist Site Mapping and Layout Planning in Petrochemical Construction. Autom. Constr..

[113] Xu Y., Zhang J. (2022). UAV-Based Bridge Geometric Shape Measurement Using Automatic Bridge Component Detection and Distributed Multi-View Reconstruction. Autom. Constr..

[114] Bosché F. (2010). Automated Recognition of 3D CAD Model Objects in Laser Scans and Calculation of As-Built Dimensions for Dimensional Compliance Control in Construction. Adv. Eng. Inform..

[115] Surmann H., Nüchter A., Hertzberg J. (2003). An Autonomous Mobile Robot with a 3D Laser Range Finder for 3D Exploration and Digitalization of Indoor Environments. Robot. Auton. Syst..

[116] Dawood T., Zhu Z., Zayed T. (2017). Machine Vision-Based Model for Spalling Detection and Quantification in Subway Networks. Autom. Constr..

[117] Kardovskyi Y., Moon S. (2021). Artificial Intelligence Quality Inspection of Steel Bars Installation by Integrating Mask R-CNN and Stereo Vision. Autom. Constr..

[118] Wang X., Dunston P.S. (2006). Potential of Augmented Reality as an Assistant Viewer for Computer-Aided Drawing. J. Comput. Civ. Eng..

[119] Harper C., Tran D., Jaselskis E., National Cooperative Highway Research Program, National Cooperative Highway Research Program Synthesis Program, Synthesis Program, Transportation Research Board (2019). National Academies of Sciences, Engineering, and Medicine. Emerging Technologies for Construction Delivery.

[120] Yang X., Guan J., Ding L., You Z., Lee V.C.S., Mohd Hasan M.R., Cheng X. (2021). Research and Applications of Artificial Neural Network in Pavement Engineering: A State-of-the-Art Review. J. Traffic Transp. Eng. Engl. Ed..

[121] Choi S.H., Kim M., Lee J.Y. (2018). Situation-Dependent Remote AR Collaborations: Image-Based Collaboration Using a 3D Perspective Map and Live Video-Based Collaboration with a Synchronized VR Mode. Comput. Ind..

[122] Harikrishnan A., Said Abdallah A., Ayer S.K., El Asmar M., Tang P. (2021). Feasibility of Augmented Reality Technology for Communication in the Construction Industry. Adv. Eng. Inform..

[123] Trappey A.J.C., Trappey C.V., Chao M.-H., Wu C.-T. (2022). VR-Enabled Engineering Consultation Chatbot for Integrated and Intelligent Manufacturing Services. J. Ind. Inf. Integr..

[124] Manuel Davila Delgado J., Oyedele L. (2022). Robotics in Construction: A Critical Review of the Reinforcement Learning and Imitation Learning Paradigms. Adv. Eng. Inform..

[125] Pantoja-Rosero B.G., Achanta R., Kozinski M., Fua P., Perez-Cruz F., Beyer K. (2022). Generating LOD3 Building Models from Structure-from-Motion and Semantic Segmentation. Autom. Constr..

[126] Wei S., Luo M., Zhu L., Yang Z. (2023). Using Object-Oriented Coupled Deep Learning Approach for Typical Object Inspection of Transmission Channel. Int. J. Appl. Earth Obs. Geoinf.

[127] Wang T., Gan V.J.L. (2023). Automated Joint 3D Reconstruction and Visual Inspection for Buildings Using Computer Vision and Transfer Learning. Autom. Constr..

[128] Chen P.-Y., Wu Z.Y., Taciroglu E. (2021). Classification of Soft-Story Buildings Using Deep Learning with Density Features Extracted from 3D Point Clouds. J. Comput. Civ. Eng..

[129] Wang W., Gao W., Cui H., Hu Z. (2020). Reconstruction of Lines and Planes of Urban Buildings with Angle Regularization. ISPRS J. Photogramm. Remote Sens..

[130] Zhao X., Cheah C.C. (2023). BIM-Based Indoor Mobile Robot Initialization for Construction Automation Using Object Detection. Autom. Constr..

[131] Dan H.-C., Bai G.-W., Zhu Z.-H., Liu X., Cao W. (2022). An Improved Computation Method for Asphalt Pavement Texture Depth Based on Multiocular Vision 3D Reconstruction Technology. Constr. Build. Mater..

[132] Ibrahim A., Golparvar-Fard M., El-Rayes K. (2022). Multiobjective Optimization of Reality Capture Plans for Computer Vision–Driven Construction Monitoring with Camera-Equipped UAVs. J. Comput. Civ. Eng..

[133] Jarząbek-Rychard M., Borkowski A. (2016). 3D Building Reconstruction from ALS Data Using Unambiguous Decomposition into Elementary Structures. ISPRS J. Photogramm. Remote Sens..

[134] Chung W.-Y., Lee B.G., Yang C.S. (2009). 3D Virtual Viewer on Mobile Device for Wireless Sensor Network-Based RSSI Indoor Tracking System. Sens. Actuators B Chem..

[135] Over M., Schilling A., Neubauer S., Zipf A. (2010). Generating Web-Based 3D City Models from OpenStreetMap: The Current Situation in Germany. Comput. Environ. Urban Syst..

[136] Qin R., Feng B., Xu Z., Zhou Y., Liu L., Li Y. (2021). Web-Based 3D Visualization Framework for Time-Varying and Large-Volume Oceanic Forecasting Data Using Open-Source Technologies. Environ. Model. Softw..

[137] Kim J., Chung D., Kim Y., Kim H. (2022). Deep Learning-Based 3D Reconstruction of Scaffolds Using a Robot Dog. Autom. Constr..

[138] Bolourian N., Hammad A. (2020). LiDAR-Equipped UAV Path Planning Considering Potential Locations of Defects for Bridge Inspection. Autom. Constr..

[139] Colvalkar A., Pawar S.S., Patle B.K. (2023). In-Pipe Inspection Robotic System for Defect Detection and Identification Using Image Processing. Mater. Today Proc..

[140] Halder S., Afsari K., Chiou E., Patrick R., Hamed K.A. (2023). Construction Inspection & Monitoring with Quadruped Robots in Future Human-Robot Teaming: A Preliminary Study. J. Build. Eng..

[141] Le N., Tran D., Sturgill R., Harper C. (2024). Exploring Remote Sensing and Monitoring Technology for Highway Infrastructure Inspection. Constr. Res. Congr..

[142] Karim M.M., Dagli C.H., Qin R. (2020). Modeling and Simulation of a Robotic Bridge Inspection System. Procedia Comput. Sci..

[143] Lei B., Wang N., Xu P., Song G. (2018). New Crack Detection Method for Bridge Inspection Using UAV Incorporating Image Processing. J. Aerosp. Eng..

[144] Liu D., Chen J., Hu D., Zhang Z. (2019). Dynamic BIM-Augmented UAV Safety Inspection for Water Diversion Project. Comput. Ind..

[145] Hamdan A.-H., Taraben J., Helmrich M., Mansperger T., Morgenthal G., Scherer R.J. (2021). A Semantic Modeling Approach for the Automated Detection and Interpretation of Structural Damage. Autom. Constr..

[146] Mandirola M., Casarotti C., Peloso S., Lanese I., Brunesi E., Senaldi I. (2022). Use of UAS for Damage Inspection and Assessment of Bridge Infrastructures. Int. J. Disaster Risk Reduct..

[147] Melo R.R.S.D., Costa D.B., Álvares J.S., Irizarry J. (2017). Applicability of Unmanned Aerial System (UAS) for Safety Inspection on Construction Sites. Saf. Sci..

[148] Messi L., García de Soto B., Carbonari A., Naticchia B. (2022). Spatial Conflict Simulator Using Game Engine Technology and Bayesian Networks for Workspace Management. Autom. Constr..

[149] Mirhosseini M., Fazlali M., Tabatabaee Malazi H., Izadi S.K., Nezamabadi-pour H. (2021). Parallel Quadri-Valent Quantum-Inspired Gravitational Search Algorithm on a Heterogeneous Platform for Wireless Sensor Networks. Comput. Electr. Eng..

[150] Monji-Azad S., Kinz M., Hesser J., Löw N. (2023). SimTool: A Toolset for Soft Body Simulation Using Flex and Unreal Engine. Softw. Impacts.

[151] Svensgaard J., Jensen S.M., Christensen S., Rasmussen J. (2021). The Importance of Spectral Correction of UAV-Based Phenotyping with RGB Cameras. Field Crops Res..

